# New insights into the anti-erosive property of a sugarcane-derived cystatin: different vehicle of application and potential mechanism of action

**DOI:** 10.1590/1678-7757-2021-0698

**Published:** 2022-07-22

**Authors:** Carlos Condarco GIRONDA, Vinícius Taioqui PELÁ, Flávio HENRIQUE-SILVA, Alberto Carlos Botazzo DELBEM, Juliano Pelim PESSAN, Marília Afonso Rabelo BUZALAF

**Affiliations:** 1 Universidade de São Paulo Faculdade de Odontologia de Bauru Departamento de Ciências Biológicas Bauru SP Brasil Universidade de São Paulo, Faculdade de Odontologia de Bauru, Departamento de Ciências Biológicas, Bauru, SP, Brasil.; 2 Universidade Federal de São Carlos Departamento de Genética e Evolução São Carlos SP Brasil Universidade Federal de São Carlos, Departamento de Genética e Evolução, São Carlos, SP, Brasil.; 3 Universidade Estadual Paulista Faculdade de Odontologia de Araçatuba Departamento de Odontologia Preventiva e Restauradora SP Brasil Universidade Estadual Paulista, Faculdade de Odontologia de Araçatuba, Departamento de Odontologia Preventiva e Restauradora, SP, Brasil.

**Keywords:** Acquired pellicle, Tooth erosion, Cystatin

## Abstract

A new sugarcane-derived cystatin (CaneCPI-5) showed anti-erosive properties when included in solutions and strong binding force to enamel, but the performance of this protein when added to gel formulations and its effect on surface free energy (SFE) requires further studies.

**Objective:**

1) to evaluate the protective effect of gels containing different concentrations of CaneCPI-5 against initial enamel erosion (Experiment 1); and 2) to analyze the SFE (γ_S_) after treating the enamel surface with CaneCPI-5 solution (Experiment 2).

**Methodology:**

In Experiment 1, 75 bovine enamel specimens were divided into five groups according to the gel treatments: placebo (negative control); 0.27%mucin+0.5%casein (positive control); 0.1 mg/mL CaneCPI-5; 1.0 mg/mL CaneCPI-5; or 2.0 mg/mL CaneCPI-5. Specimens were treated with the gels for 1 min, the AP was formed (human saliva) for 2 h and the specimens were incubated in 0.65% citric acid (pH=3.4) for 1 min. The percentage of surface hardness change (%SHC) was estimated. In Experiment 2, measurements were performed by an automatic goniometer using three probing liquids: diiodomethane, water and ethylene glycol. Specimens (n=10/group) remained untreated (control) or were treated with solution containing 0.1 mg/mL CaneCPI-5, air-dried for 45 min, and 0.5 µL of each liquid was dispensed on the surface to measure contact angles.

**Results:**

Gels containing 0.1 and 1.0 mg/mL CaneCPI-5 significantly reduced %SHC compared to the other treatments (p<0.05). Treated enamel showed significantly lower γ_S_ than control, without changes in the apolar component (γ_S_^LW^), but the polar component (γ_S_^AB^=Lewis acid-base) became more negative (p<0.01). Moreover, CaneCPI-5 treatment showed higher γ_S_^-^ (electron-donor) values compared to control (p<0.01).

**Conclusions:**

Gels containing 0.1 mg/mL or 1.0 mg/mL CaneCPI-5 protected enamel against initial dental erosion. CaneCPI-5 increased the number of electron donor sites on the enamel surface, which may affect AP formation and could be a potential mechanism of action to protect from erosion.

## Introduction

Dental erosion is the chemical loss of mineralized tooth substance due to exposure to non-bacterial acids.^1^ Saliva is the main patient-related factor that interferes with dental erosion, since it is saturated regarding apatite, buffers the acids and is the main source of proteins that form the acquired pellicle (AP). This proteinaceous layer acts as a mechanical barrier to the acids, thus reducing erosion.^2^

Not all proteins found in the AP protect the tooth surface from acid dissolution. Studies have suggested that the proteins present in the basal layer have a greater participation in this regard.^3^ Thus, the concept of “acquired pellicle engineering”, which involves changing the AP by adding molecules, has a strong potential to increase its protective effect on the tooth surface.^4 , 5^ A series of *in vivo* studies used proteomic approaches to identify acid-resistant proteins in the AP that would be candidates for inclusion in dental products to reduce erosive demineralization.^6 - 8^ Among them, cystatin-B is a good alternative,^6^ but the cost of the human recombinant protein is prohibitive. Therefore, our group recently cloned sugarcane-derived cystatin (CaneCPI-5) that has a strong binding force to hydroxyapatite and can protect from initial erosion *in vitro* ^4^ and *in vivo* ^5^ when added to rinse solutions. Moreover, incorporating molecules in the AP may change the enamel reactivity and the surface free energy (SFE), which might guide protein binding to the AP, thus changing its composition.

Regarding the application vehicle, the use of gels in studies involving the inhibition of matrix metalloproteinases in dentin a offered better protection against dentin erosion when compared with their inclusion in solutions.^9 , 10^ Possibly due to the prolonged contact time of the gel with the tooth surface, due to its viscosity. We hypothesize that the same could happen with CaneCPI-5 gels. If the protection conferred by CaneCPI-5-containing gels is better than that conferred by solutions, the frequency of application of the first can be lower, which is an advantage from the clinical point of view.

Therefore, our study evaluates the protective effect of gels containing different concentrations of CaneCPI-5 against enamel initial erosion *in vitro* . Since little is known about the mechanisms by which CaneCPI-5 interacts with the enamel surface, we also analyzed the ability of CaneCPI-5 to alter the SFE of enamel by measuring the contact angle using the sessile drop method. The null hypotheses tested were: 1) gels containing CaneCPI-5 do not protect from initial dental erosion and 2) CaneCPI-5 does not alter the enamel SFE.

## Methodology

This study comprised two experiments: In Experiment 1, the effect of CaneCPI-5 (in different concentrations) added in gel on polished enamel specimens was evaluated using surface microhardness analysis. In Experiment 2, 0.1 mg/mL CaneCPI-5 (in solution) was applied on polished enamel specimens for SFE analysis.

The use of bovine teeth for this research was approved by the Ethics Committee on Animal Use of Bauru School of Dentistry, University of São Paulo (Protocol: 006/2017 for Experiment 1 and Protocol: 010/2021 for Experiment 2). Also, this study was approved by the Ethics Committee for Human Research (CAAE: 59786416.9.0000.5417) of Bauru School of Dentistry, University of São Paulo. Besides, saliva donor volunteers signed an informed consent form before the procedures.

### Selection of volunteers and saliva collection

Saliva was collected from three healthy volunteers of both genders (aged 24 to 32 years). The exclusion criteria adopted were: smoking habit, cavitated carious lesions, severe dental wear, use of medications that affect salivary flow, salivary flow under the thresholds for unstimulated (> 0.3 mL/min) and stimulated (> 1.0 mL/min) saliva, xerostomia, type I diabetes, poor nutrition, gastroesophageal problems and regurgitation and vomiting disorders.^11^

All volunteers performed oral hygiene before collection using a new toothbrush, fluoride toothpaste (CloseUP, 1450 ppm F, Unilever, Brazil) and dental floss. Saliva was collected between 9 and 11 a.m. (to avoid circadian effects) under masticatory stimulation using Parafilm. Then, saliva was centrifuged (14,000 g at 4ºC) for 15 min. After that, the supernatants were collected to form a pool of saliva and divided into 13-mL aliquots, which were stored at -80°C prior to the experiments.^12^

### Heterologous expression of CaneCPI-5

CaneCPI-5 was produced at the Laboratory of Molecular Biology of the Department of Genetics and Evolution of the Federal University of São Carlos, Brazil. For heterologous expression, bacterial strain *Escherichia coli Rosetta* (DE3) transformed with plasmid pET28aCaneCPI-5 was used as previously described.^13^ The expressed protein was purified from the soluble fraction of bacterial cultures induced by IPTG (isopropyl-beta-D-thiogalactoside), subjected to centrifugation and sonication. The purification was done by affinity chromatography using columns containing Ni-NTA Superflow nickel resin (Qiagen).^13^

### Preparation of the enamel specimens

A total of 95 bovine enamel specimens were prepared (4 mm×4 mm×4 mm), being 75 specimens for “Experiment 1” and 20 specimens for “Experiment 2”. They were obtained from the buccal-cervical region of bovine incisors and stored in 2% thymol solution (pH 7.0) for 30 days. Besides, the specimens were visually analyzed to assess possible stains and cracks. In these cases, the teeth were excluded. Then, the enamel surface was sequentially polished using water-cooled silicon carbide paper disks (320, 600, and 1200 grit, Extec, Enfield, CT, USA). A felt polishing cloth (Extec Corp. Polishing cloth; Buehler, Lake Bluff, IL, USA), moistened with a 1-μm diamond solution (Extec Corp. Buehler, Lake Bluff, IL, USA), was used on the surface of interest to finalize the polishing. After polishing, the specimens were immersed in an ultrasonic bath (T7 Thornton, Unique Ind. E Com. Ltda., São Paulo, SP, BR) with deionized water for seven min at 25°C. Lastly, they were stored (with wet gauze) at 4°C prior to the experiment.

### Experiment 1. Effect of gels containing different concentrations of CaneCPI-5 against initial enamel erosion *in vitro*

#### Experimental procedures

A total of 75 specimens were divided into five groups (n/group=15, determined by computerized random numbers after initial surface hardness): 1) placebo gel (negative control), 2) 0.27% mucin + 0.5% casein (positive control), 3) 0.1 mg/mL CaneCPI-5, 4) 1 mg/mL CaneCPI-5 and 5) 2.0 mg/mL CaneCPI-5. All gels were prepared as described by Kato, et al.^9^ (2010) and had the same composition, except for the presence of casein + mucin or CaneCPI-5.

The amount of gel applied was controlled by a dispenser (pipette, 20 µl per specimen), then the gel was added on the microbrush and applied on the enamel surface of each specimen for 1 minute, and the excess was removed with a cotton swab.^9^ The specimens were then incubated in saliva for 2 h at 37°C under agitation to form the AP.^14^ Then, the specimens were washed in deionized water (10 s) and air-dried (5 s). For the erosive challenge, they were immersed in 0.65% citric acid solution (pH=3.4) for 1 min at 30°C under agitation, washed in deionized water and air-dried again.^14^

#### Surface hardness

Surface hardness change (SHC) analyses were performed using a Knoop penetrator, with a load of 50 g for 15 s at baseline (SHC_initial_) and after the experiment (SHC_final_). Five indentations were made in the central region of each specimen at 50 μm intervals. Control indentations of 2 and 5 g were made to detect possible loss of surface. Specimens with microhardness values 10% lower or 10% higher than the mean of all specimens were excluded from the study. The percentage of surface hardness change (%SHC) was estimated as a measure of enamel softening, according to the following equation: $\%\mathrm{SHC}=([{\mathrm{SHC}}_{\mathrm{initial}}–{\mathrm{SHC}}_{\mathrm{final}}]/\;{\mathrm{SHC}}_{\mathrm{initial}})\times 100^{4}$ .

## Experiment 2. Ability of CaneCPI-5 to alter the enamel surface free energy

Twenty enamel specimens were divided into two groups, as follows: Negative control (untreated) or 0.1 mg/mL CaneCPI-5 (n=10/group determined by computerized random numbers).

### Surface free energy measurements

The surface free energy (SFE) was characterized by contact angle measurements, using the sessile drop method to determine the SFE. Measurements were performed by an automatic goniometer (DSA 100S, Krüss, Hamburg, Germany) using three probing liquids: diiodomethane, water and ethylene glycol. The treated specimens were air dried for 45 min to stabilize the layer formed.^15^ Then, 0.5 µL of each liquid was dispensed on the surface of each block and the contact angles were measured using the images captured by a CCD camera. Five measurements were performed at 20°C and relative air humidity of 47% for each specimen.^15 , 16^ Different parameters, such as acid (g^+^, receptor component), base (g^-^, donor component) and Lifshiz van der Waals (g^LW^, nonpolar component) of surface free energy (mN/m) were estimated according to the model of van Oss, Chaudhery and Good to determine the substrates free energy.^17 , 18^ The interaction free energy (D *G*_iwi_) was also estimated to determine the hydrophobicity/hydrophilicity of the enamel surface: D *G*_iwi_ > 0 indicated a hydrophilic surface and D *G*_iwi_ < 0 indicated a hydrophobic surface.^16 , 19^

## Statistical Analysis

All the data were analyzed using the GraphPad InStat (version 3.10 for Windows) and GraphPad Prism (GraphPad Software Inc., La Jolla, CA) software. Data were checked for normality (Kolmogorov-Smirnov test) and homogeneity (Bartlett test) to select the appropriate statistical test. In the first experiment, the data were analyzed using Kruskall-Wallis and Dunn’s tests. In the second experiment, the data were analyzed using ANOVA and Student-Newman-Keuls’s test and by Pearson’s correlation coefficient. The significance levels of both experiments were considered as p<0.05.

## Results

**Figure 1:**
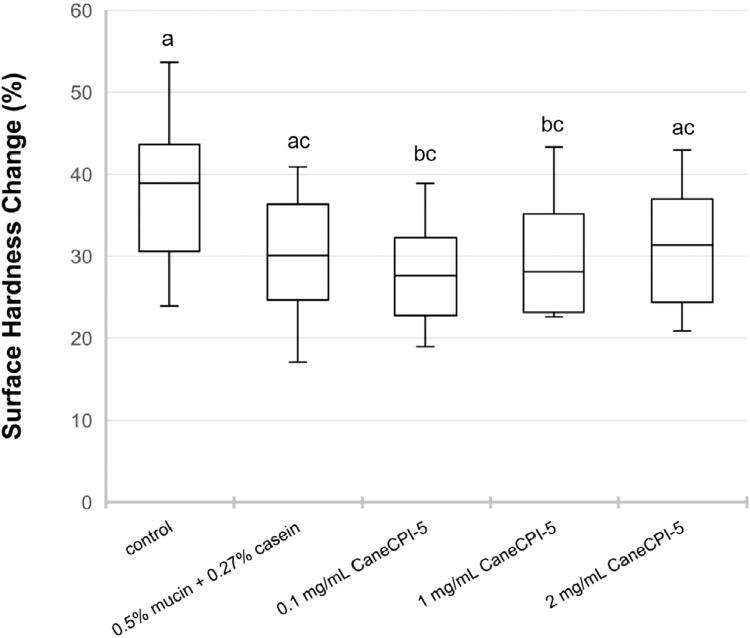
Median enamel loss after short-term erosive challenge. Bovine enamel specimens were treated with gels containing the proteins for 1 min, followed by incubation in pooled human saliva for 2 h to form the acquired pellicle and subsequent challenge with 0.65% citric acid for 1 min. Different letters indicate a significant difference among groups (Kruskal-Wallis and Dunn’s tests, p<0.05, n=15/group)

In the first experiment, only the treatments with CaneCPI-5 at 0.1 and 1.0 mg/mL significantly reduced the SHC compared to control (p<0.05). The treatment performed with the higher concentration of CaneCPI-5 did not significantly differ from control or from mucin + casein (p>0.05) ( Figure 1 ).

**Figure 2:**
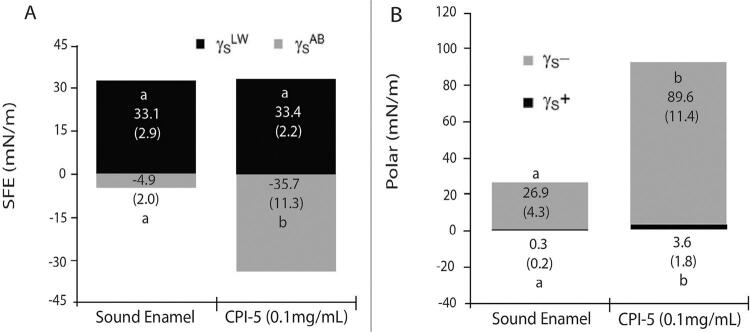
(A) Surface free energy and their components (γ *S* LW: Lifshitz-van der Waals surface tension component; γ *S* AB: Lewis acid-base interaction) with different enamel-surface treatments. (B) Influence of the treatments on the polar component of surface free energy on enamel surface: Lewis-acid (γS+) and Lewis-base (γS−). Values denote mean and standard deviation (n=10). Distinct letters show significant differences among mean considering treatment (Student-Newman-Keuls, p<0.05)

In the second experiment, the SFE (γ_S_) was significantly lower with CaneCPI-5 (p<0.001) compared to control ( Table 1 ; p<0.001). The values of the apolar component (γ_S_^LW^) from enamel surface were not significantly different between the groups (p=0.161). The values of the polar component (γ_S_^AB^=Lewis acid-base) became more negative with CaneCPI-5 treatment ( Figure 2A ; p<0.001). Among the parameters from γ_S_^AB^, γ_S_^+^=electron-acceptor (Lewis acid) and γ_S_^-^=electron-donor (Lewis base), the CaneCPI-5 treatment showed higher γ_S_^−^ values compared to control ( Figure 2B ; p<0.001). We observed significant correlations between γ_S_ and γ_S_^AB^ values (Pearson’s r=0.987; p<0.001) and γ_S_^−^ (Pearson’s r=-0.942; p<0.001). The interaction free energy (D *G*_iwi_) was > 0 for CaneCPI-5 treatment, indicating a hydrophilic surface ( Table 1 ).

**Table 1:** Means (SD) of the contact angles of probing liquids, surface free energy (γS) and interaction free energy (D *G* iwi) after treating enamel surface with 0.1 mg/mL CaneCPI-5 or not (n=10).

Treatments	Water	Diiodomethane	Ethylene glycol	γS	Δ *G*_iwi_
	q (°)	q (°)	q (°)	(mN/m)	(mN/m)
Sound enamel	67.3^a^	52.6^a^	56.6^a^	28.2^a^	-0.3^a^
	(-4.4)	(-5.1)	(-3.6)	(-4.5)	(-8.2)
CPI-5 (0.1 mg/mL)	37.2^b^	51.6^a^	55.9^a^	-1.7^b^	53.2^b^
	(-2.8)	(-3.8)	(-4.3)	(-10.8)	(-5.6)

Distinct superscript letters indicate significant difference among the groups in each analysis (ANOVA and Student-Newman-Keuls's test, p<0.05, n=10)

## Discussion

Our study involves the concept of “acquired pellicle engineering” that involves changing the AP by adding molecules or ions that can increase its protection against dental erosion.^20^ The change was done by using CaneCPI-5, a sugarcane-derived cystatin that has a strong binding force to hydroxyapatite.^4^

In Experiment 1, we used an established initial erosion model to evaluate the protective effect of a gel containing CaneCPI-5 against erosion. This model involves one challenge (1 min) with 0.65% citric acid (pH 3.5),^14^ causing enamel softening that can be measured by SHC, since enamel loss (detected by profilometry) is not expected at this early stage. Our previous studies showed protective effect in applying solutions containing 0.1 mg/mL CaneCPI-5 against initial enamel erosion when applied *in vitro* for two h^4^ or *in vivo* for 1 min^5^ . Furthermore, the use of gels containing protease inhibitors^9^ offered better protection against dentine erosion than solutions containing the same inhibitors,^10^ due to a more intimate contact of the gels with the dental surfaces. We used a mixture of mucin (0.27%) and casein (0.5%) as a positive control due to the previous results, which showed that adding both components in the AP could offer a protective effect against initial erosion *in vitro.* ^14^

Thus, this vehicle was selected for application of CaneCPI-5, at concentrations (ranging between 0.1 and 2.0 mg/mL), based on those used in solutions.^4^ The gels containing CaneCPI-5 at 0.1 and 1.0 mg/mL significantly reduced enamel erosion compared to the placebo gel, while the product containing 2.0 mg/mL CaneCPI-5 did not ( Figure 1 ). The gels offered 30%SHC reduction, whereas the effects using aqueous solutions at the same concentrations was around 90%.^4^ However, the solutions remained in contact with the enamel surface for 2 h, whereas treatment with the gels lasted only 1 min. Besides, in Atomic Force Microscopy (AFM), enamel samples were incubated in solutions containing CaneCPI-5 for 4 h.^4^ Based on time-response considerations, it would be helpful to evaluate longer exposure to the gels (e.g., 4 min), since application of fluoridated gels for 4 min in the clinical practice has been reported to offer higher caries-protective effects than application for 1 min.^21 , 22^ The highest concentration of CaneCPI-5 (2.0 mg/mL) did not protect enamel from initial erosion. This can be related to previous studies showing that sugarcane cystatins, at high concentrations, undergo dimerization by domain swapping,^23 , 24^ which reduces the levels of free protein to bind to enamel.

Experiment 2 had a mechanistic approach. We aimed to test the enamel reactivity after treatment with CaneCPI-5 using the sessile drop method. This is essential for the concept of “acquired pellicle engineering”, since alterations in the SFE upon treatment with CaneCPI-5 might guide protein binding to the AP, thus changing its composition, especially considering binding other salivary proteins to CaneCPI-5 and/or to dental surfaces. The untreated enamel was slightly hydrophobic, since contact water angle was a little larger than 65°;^25 , 26^ SFE (γ_s_) was < 30 mN/m ( Table 1 ),^19 , 27^ D *G*_iwi_ was close to zero, and γ_s_^−^ was < 28.5 mN/m ( Figure 2 ),^16 , 19 , 28^ with values of γ_s_^+^ close to zero. As described in a previous study,^29^ enamel surface shows characteristics that favor the precipitation of ionic species, such as Ca^2^ and CaH_2_PO_4_^+^, or protein adhesion, both of which are essential to reduce the erosive process. Furthermore, surfaces with lower SFE brings fewer bacteria to its surface than one with higher SFE. However, we emphasize that the acid-base theoretical approach used in this study,^16 , 19 , 28^ involving the decomposition in γ_s_^LW^ and γ_s_^AB^ (which strongly influence to γ_s_), differs from other studies that used different theoretical approaches to estimate γ_s_.

In our study, the reduction of SFE with CaneCPI-5 treatment was related to more negative values of polar energy (γ_s_^AB^), since the nonpolar energy did not change (γ_s_^LW^). Therefore, the acid (γ_s_^+^)/base (γ_s_^−^) and interaction free energy (D *G*_iwi_) forces indicate whether a surface is more hydrophobic or hydrophilic, facilitating or not protein adhesion or calcium phosphate precipitation.^16 , 28 , 29^ The theoretical aspects above show that treatment with CaneCPI-5 increases the hydrophilic character of the surface of the enamel, which makes it prone to water, considering contact angles < 65°, γ_s_^−^ > 28.5 mN/m and D *G*_iwi_ > 0^30^ . Also, CaneCPI-5 showed higher γ_s_^−^ values, leading to higher electron-donor sites at the enamel surface that favors adsorption cationic ionic species (Ca^2^ and CaH_2_PO_4_^+^) and cationic acid-resistant proteins from saliva, thus explaining the lower hardness loss after erosion challenge. Consequently, negative surfaces may be partially or fully neutralized by multivalent cations, leading to a hydrophobic surface.^31^ Alteration in the SFE partially explains the changes in acid-resistant proteins of the AP obtained after rinsing for 1 min with 0.1 mg/mL CPI-5 and subsequent challenge with 1% citric acid pH 2.5 for 10 s (increase in keratin, IgG, lactotransferrin, serum albumin, alpha amylase, basic salivary proline-rich protein, carbonic anhydrase).^5^

We recognize the limitations of the present *in vitro* study. Although the protocols suit preliminary studies, they do not accurately simulate the clinical condition due to the absence of oral cavity-specific factors, such as the formation of AP. In Experiment 1, limitation of treatment time (with the gels and CaneCPI-5) for 1 min could be extended for longer periods (e.g., 4 min). Regarding Experiment 2, the presence of saliva, which is the main biological factor involved in the occurrence of dental erosion whose factor is the most determinant for the oral cavity, was not considered. Moreover, CaneCPI-5 was included in gels in the first experiment, while it was included in solution in the second one, due to the analytical technique used. These limitations must be addressed in future studies.

We rejected both hypotheses based on the results, since: 1) gels containing CaneCPI-5 at 0.1 and 1.0 mg/mL protected enamel from initial dental erosion; and 2) CaneCPI-5 altered the enamel SFE. Moreover, change in SFE of enamel after applying CaneCPI-5 may help to partially explain alterations in the AP proteome, with consequent change in its protective ability, induced by this phytocystatin.
